# Gender differences in treatments and interventions received by children and adolescents with cerebral palsy

**DOI:** 10.1186/s12887-020-1926-4

**Published:** 2020-01-30

**Authors:** Annika Lundkvist Josenby, Tomasz Czuba, Ann I. Alriksson-Schmidt

**Affiliations:** 1 Physiotherapy Department, Children’s Hospital, Skåne University Hospital, Lund, Sweden; 2 Department of Health Sciences, Lund University, Lund, Sweden; 3 Department of Clinical Sciences, Lund University, Lund, Sweden; 4 Department of Clinical Sciences, Lund University, Skåne University Hospital, Orthopedics, Lund, Sweden

**Keywords:** Cerebral palsy, Treatment, Gender, Sex, Physiotherapy, Occupational therapy

## Abstract

****Background**:**

In the Swedish population-based follow-up program and national quality registry for individuals with cerebral palsy (CPUP), physiotherapy (PT) and occupational therapy (OT) treatments are regularly recorded along with functional status. By Swedish law, all citizens irrespective of personal characteristics or socioeconomic status, have the right to receive healthcare and medical treatments as applicable. Previous research has shown gender differences in treatments and interventions received by children with cerebral palsy (CP). The purpose of this study was to examine differences in treatments and interventions by gender and place of birth in children and adolescents participating in CPUP.

****Methods**:**

This was a cross-sectional registry study. Data from the latest PT (*n* = 2635) and OT assessment forms (*n* = 3480) in CPUP were extracted for individuals aged 0–17 years. Logistic regressions were used to assess the relationships between the outcome variables and gender and place of birth (including an interaction term gender X place of birth), adjusted for age, Gross Motor Function Classification System (GMFCS) levels and spasticity scores for PT interventions and Manual Ability Classification System (MACS) for OT interventions.

****Results**:**

Results are presented as odds ratios [95% confidence intervals] and *p*-values. Girls were significantly more likely to have spinal braces than boys; 1.54 [1.07, 2.22] *p* < 0.05, a significant interaction with place of birth indicated fewer spinal braces prescribed to children born outside of the Nordic countries; 0.20 [0.079, 0.53] *p* < 0.001. Girls were less likely to have undergone selective dorsal rhizotomy (SDR); 0.49 [0.25, 0.94] *p* < 0.05. Individuals born outside of the Nordic countries, were significantly less likely to have received intrathecal baclofen (ITB) 0.27 [0.074, 0.98] *p* < 0.05.

****Conclusions**:**

Of the treatments prescribed, gender differences were observed for spinal braces and having undergone SDR. A statistically significant difference based on place of birth was noted for spinal bracing and having received ITB treatment. Other PT and OT treatments were associated with age, level of spasticity, and functional severity as classified using the GMFCS and the MACS. Increased awareness of differences based on gender, and where a child is born, could be obtained by inter- and intraprofessional discussions.

## Background

Healthcare services should be needs and evidence-based. However, a variety of treatment modalities might be available for the same condition and scientific evidence does not always exist. Macro factors (e.g., national healthcare policies) and contextual factors (e.g., available resources, staff shortages) affect what treatments are provided. Insurance plans might reimburse a limited number of treatments, and countries with universal healthcare may be hampered by long waiting lists and geographical differences in availability. The decision-making processes in healthcare are influenced by human beliefs and behaviors. To prescribe and adhere to treatments and interventions require concerted actions by both healthcare providers and patients [1]. In the case of minors and those cognitively unable to make their own decisions, caretakers play an integral part. Humans operate in accordance with their preconceived notions, opinions and biases—conscious or unconscious —which may affect who is offered certain treatments or interventions [2, 3]. For providers, the type of training received, and exposure to certain “clinic cultures” and traditions influence treatment recommendations. Moreover, providers may be influenced by factors such as the gender, age and ethnicity of the patients [4, 5]. Caregivers might also have different expectations for their children, depending on the gender of the child. This may result in unwarranted differences in the treatment strategies endorsed, and, by extension, health disparities.

Bias is highly relevant also in the context of disability. Cerebral palsy (CP) is an umbrella term describing a life-long disability that negatively affects motor function. It is caused by non-progressive brain injuries that occur in utero or early on in life [6, 7]. Great variability in gross- and fine motor functions, cognitive functions, and communication abilities exist in this population [6], and comorbidities and secondary conditions frequently co-occur [7, 8]. For decades, the prevalence of CP remained stable at 2–3 per 1000 live births in the Western world [9–12]. However, recently multinational data have shown a decreasing trend in the prevalence of CP [13]. Functions and abilities in CP can be classified according to a number of classification schemes. The most commonly used are based on five-level ordinal classification structures, where level I indicates that the function/ability is the least affected and level V the most affected. The Gross Motor Function Classification System (GMFCS) [14], the Manual Abilities Classification System (MACS) [15], and the Communication Function Classification System (CFCS) [16] are the most commonly used [17].

In Sweden, approximately 95% of children and adolescents with CP born 2000 or later participate in the Cerebral Palsy Follow-Up Program (CPUP) [18]. CPUP is a population-based joint follow-up program and national registry implemented at all habilitation units nationwide. In CPUP, there is a slight male predominance at all levels of GMFCS (with the exception of level II), at all levels of MACS and for all CP subtypes (with the exception of ataxic CP), which is in line with corresponding data from other national registries [19–21]. In two recently published studies based on CPUP data, study authors noted gender differences on treatments received [22, 23]. One of the studies included CPUP data from five northern counties in Sweden, and based on univariate analyses, in three of the five counties girls received less physiotherapy (PT) and less often than boys. Furthermore, boys participated more frequently in physical education than girls [23]. The other study included CPUP data from all of Sweden, and results showed that boys were significantly more likely than girls to have received botulinum toxin A (BTX-A) since their last CPUP-assessment [22]. The gender of the caregivers and children have been reported by other researchers not to affect the caregivers’ treatment acceptability of BTX-A injections in children with CP [24]. These findings raise at a minimum two pertinent questions; what explain the gender differences in treatments observed, and are there other gender differences in treatments received in children and adolescents with CP?

## Methods

### Aim, design, and setting

The aim of this cross-sectional registry study was to compare and explore the use of therapies and interventions prescribed to children with CP in Sweden, and if these differed by the gender of the child. We also explored if there were differences in treatments received based on if the children were born in the Nordic countries. The focus was on interventions and treatments because these are modifiable in that somebody (provider, caregiver, and/or individual) has made active decisions to prescribe or adhere to specific treatments. Importantly, the aim was not to determine if boys or girls received *better* treatment (more is not per definition better, and in some cases, treatments may even be harmful) but to assess and explain differences in treatments received between genders, if applicable.

The legal caregivers provide oral consent prior to participation in CPUP, and the children provide verbal assent, as applicable. Participation can be discontinued at any time, and the decision to withdraw will not affect the healthcare received. The Regional Ethical Review Board in Lund, Sweden (443–99, revised 2009) approved the study.

### Characteristics of participants and description of materials

All re/habilitation units in Sweden where children with CP receive care participate in CPUP, and data were retrieved from the most recent PT and OT assessments in the years 2016 and 2017. Children suspected of having CP are eligible to participate, resulting in a population-based database that includes approximately 95% of children with (or suspected) CP born after the year 2000 [25]. The program is multidisciplinary and involves professionals from specialties such as orthopedic surgery, pediatric neurology, hand surgery, occupational therapy (OT) and PT. CPUP, or a modified version of the program, has been implemented in Norway, Denmark, Iceland, Scotland, New South Wales (Australia), and most recently in Jordan [25, 26]. Participants follow assessment schedules, and based on age and GMFCS level, they complete CPUP assessments once or twice per year. PTs and OTs perform the bulk of the CPUP assessments. These two disciplines are responsible for different parts of the CPUP assessments and the data are entered separately into different forms in the CPUP registry. The characteristics of the participants and the distributions of the measures are therefore presented separately for PT and OT treatments and interventions (Tables 1, 2, 3 and 4).

In total, 2635 participants were included in the analyses on PT related treatments and interventions. The majority, 1528 (58%) were boys and 1107 (42%) were girls, 84.6% were born in a Nordic country (Sweden, Norway, Denmark and Finland). The mean age for boys was 9.5 (SD = 4.24) years and 9.7 (SD = 4.37) years for girls. Furthermore, 3480 participants were included in the analyses on OT related treatments and interventions. The majority 2014 (58%) were boys and 1466 (42%) were girls, 83.8% were born in a Nordic country. The mean age for boys was 9.7 (SD = 4.37) years and 9.8 (SD = 4.43) years for girls. Separate analyses were performed for PT and OT reported treatments, where reports on 2516 individuals overlapped and 1082 did not overlap. Of the 1082 non-overlapping reports, 963 were PT and 119 were OT reports.

From the PT assessment form, the following dichotomous treatment modalities (yes/no) were included in the study: use of spinal brace, received BTX-A in the lower extremities since the last CPUP-assessment, treated with spasticity reducing medication, Intrathecal Baclofen (ITB), Selective Dorsal Rhizotomy (SDR) surgery, assistive devices for supported standing, and additional PT intervention since the last CPUP assessment. Gross motor function was classified by the PTs according to the GMFCS Expanded and Revised version, which consists of five levels, where GMFCS-level I signifies the highest gross motor functional level and GMFCS-level V the most severely restricted. The GMFCS has been found to have good validity and reliability [27–29] and it has been reported to be stable over time [30]. Spasticity was assessed in the lower limbs with the modified Ashworth scale [31] by the participant’s PT. Ashworth scores from plantar flexors, knee flexors, and hip abductors were combined into a spasticity score for each individual and were used in the analyses as a general measure of increased muscle tone in the lower limbs (Table 1).

**Table 1:** Characteristics and distributions of physiotherapy related interventions in children with cerebral palsy

Variable, *n* = available data	Boys, *n* (%)	Girls, *n* (%)	Total, *n* (%)
Physiotherapy assessment form, *N* = 2635			
Place of birth, *n* = 2635			
Nordic country	1305 (85.4)	924 (83.5)	2229 (84.6)
Outside of the Nordic countries	223 (14.6)	183 (16.5)	406 (15.4)
Gross Motor Function Classification System levels, n = 2635			
I	699 (45.7)	508 (45.9)	1207 (45.8)
II	263 (17.2)	190 (17.2)	453 (17.2)
III	156 (10.2)	113 (10.2)	269 (10.2)
IV	223 (14.6)	150 (13.6)	373 (14.2)
V	187 (12.2)	146 (13.2)	333 (12.6)
Spasticity score, mean (SD), range, *n* = 2534	18.75 (16.29), 0–95.83	17.68 (15.15), 0–87.5	18.30 (15.83), 0–95.83
Received botulinum toxin A in the lower extremities since the last CPUP*assessment, *n* = 2500			
Yes	304 (21.0)	187 (17.8)	491 (19.6)
Intrathecal baclofen (pump), *n* = 2343			
Yes	38 (2.8)	15 (1.5)	53 (2.3)
Spasticity reducing medication (oral), *n* = 2467			
Yes	103 (7.2)	74 (7.1)	177 (7.2)
Selective dorsal rhizotomy, *n* = 1944			
Yes	45 (4.0)	15 (1.9)	60 (3.1)
Received physiotherapy treatment since last CPUP* assessment			
Yes	1076 (72.5)	785 (73.0)	1861 (72.7)
Use of spinal brace, *n* = 2550			
Yes	124 (8.4)	100 (9.4)	224 (8.8)
Assistive device to facilitate standing, *n* = 2423			
Yes	421 (30.1)	308 (30.1)	729 (30.1)

* National registry and cerebral palsy follow-up program (CPUP)

From the OT assessment form, the following dichotomous treatment modalities (yes/no) were included: use of orthoses in the upper extremities, received additional OT interventions (hand training or activity of daily living training) since the last CPUP assessment or in the last year, and BTX-A in the upper extremities. The OT recorded the MACS level for each child, describing manual function as one of five levels of how children with CP use their hands in daily activities. At level I, the child uses his/her hands easily and successfully and at level V the child does not handle objects and has severe difficulties in performing simple actions [15]. The distributions of the characteristics of the participants and the measures are summarized, by gender, in Table 2.

**Table 2:** Characteristics and distributions of occupational therapy related interventions in children with cerebral palsy

Variable, *n* = available data	Boys, *n* (%)	Girls, *n* (%)	Total, *n* (%)
Occupational therapy assessment form, *N* = 3480			
Place of birth, n = 3480			
Nordic country	1701 (84.5)	1214 (82.8)	2915 (83.8)
Outside of the Nordic countries	313 (15.5)	252 (17.2)	565 (16.2)
Manual Ability Classification System levels, *n* = 3397			
I	608 (30.8)	458 (32.1)	1066 (31.4)
II	503 (25.5)	335 (23.5)	838 (24.7)
III	304 (15.4)	219 (15.4)	523 (15.4)
IV	234 (11.9)	168 (11.8)	402 (11.8)
V	323 (16.4)	245 (17.2)	568 (16.7)
Received botulinum toxin A in the upper extremities since the last CPUP* assessment, n = 3480			
Yes	164 (8.1)	108 (7.4)	272 (7.8)
Orthoses, upper extremities, n = 3480			
Yes	330 (16.4)	254 (17.3)	584 (16.8)
Received occupational therapy treatment since last CPUP* assessment, *n* = 3149			
Yes	670 (36.7)	453 (34.2)	1123 (35.7)

* National registry and Cerebral Palsy follow-up program (CPUP)

### Statistical analysis

Logistic regression models (presented as odds ratio (OR) with 95% confidence intervals (CIs)) were used to assess the relationship of the outcome variables and gender, adjusted for place of birth, age, GMFCS, MACS (for OT interventions) and spasticity scores (for PT interventions). Place of birth was dichotomized into in- or outside of the Nordic countries. The spasticity score was created based on the modified Ashworth scale scores for plantar flexors, knee flexors and hip adductors (both left and right sides) where ‘1+’ assessment was counted as 1.5. The scores were summarized and divided by the maximum total score possible based on the number of sites where a score was available. For example, a child with spasticity assessments on all six sites could get a maximum score of 24 (4 points on each site), whereas a child with assessments on three sites could receive a maximum of 12 (children with more than three missing sites were excluded from the analysis). The scores were presented as percentage of the maximum possible score and the presented odds refer to 1% change in score. The analyses were performed using Stata SE 15.1 [32].

## Results

The analyses were performed and are presented separately for the two samples. Six logistic regressions were performed for PT related treatments and interventions and three logistic regressions for OT related treatments and interventions respectively. The ORs and 95% CIs from the logistic regressions are presented in Tables 3 and 4.

**Table 3:** Exponentiated coefficients; 95% confidence intervals in brackets of the physiotherapy related treatment modalities

Outcome variables (yes/no)							
Independent variables (reference group)	Use of spinal brace	Received BTX- A since last assessment	Has ITB treatment †	Oral spasticity reducing medication	Undergone SDR surgery	Assistive device for standing	Physiotherapy treatment since last assessment
Gender (female)	1.54^*^	0.81	0.49	0.96	0.49^*^	1.13	1.16
	[1.07,2.22]	[0.64,1.02]	[0.22,1.08]	[0.65,1.43]	[0.25,0.94]	[0.80,1.60]	[0.93,1.43]
Place of birth (outside of the Nordic countries)	1.49	0.70	0.27^*^	0.78	0.56	0.66	0.94
	[0.87,2.55]	[0.47,1.05]	[0.074,0.98]	[0.43,1.43]	[0.19,1.71]	[0.38,1.15]	[0.64,1.37]
Gender X place of birth	0.20^***^	1.48	0.85	0.77	1.08	0.66	0.64
	[0.079,0.53]	[0.81,2.70]	[0.068,10.6]	[0.29,2.02]	[0.16,7.20]	[0.30,1.50]	[0.37,1.11]
Age	1.05^*^	0.98	1.28^***^	1.05^*^	1.04	0.10	0.90^***^
	[1.01,1.09]	[0.96,1.00]	[1.17,1.39]	[1.01,1.09]	[0.98,1.11]	[0.96,1.03]	[0.88,0.93]
GMFCS (GMFCS I)							
II	2.77	1.17		3.57^**^	2.75^*^	15.92^***^	2.31^***^
	[0.69,11.1]	[0.86,1.58]		[1.46,8.76]	[1.12,6.76]	[5.48,46.2]	[1.77,3.01]
III	9.38^***^	1.40		4.36^**^	23.23^***^	136.2^***^	4.40^***^
	[2.78,31.68]	[0.99,2.00]		[1.72,11.07]	[11.0,49.05]	[49.05,378.4]	[2.92,6.61]
IV	76.56^***^	1.353	1	13.10^***^	4.80^**^	1001.0^***^	8.22^***^
	[27.19,215.5]	[0.97,1.90]	[1]	[5.86,29.32]	[1.85,12.50]	[355.72816.9]	[5.11,13.24]
V	222.6^***^	1.030	6.53^***^	37.29^***^	0.76	1730.4^***^	8.35^***^
	[79.16,625.7]	[0.71,1.50]	[3.11,13.68]	[16.93,82.14]	[0.10,6.02]	[594.75035.1]	[4.90,14.24]
Spasticity score	1.00	1.03^***^	1.00	1.03^***^	0.95^***^	1.02^***^	1.02^***^
	[0.99,1.01]	[1.02,1.04]	[0.98,1.02]	[1.02,1.04]	[0.93,0.97]	[1.01,1.03]	[1.01,1.03]

Relationship, presented as odds ratio with 95% confidence intervals in brackets, of the physiotherapy reported outcome variables and gender, adjusted for place of birth, age, Gross Motor Function Classification System (GMFCS) levels and spasticity score. BTX-A- Botulinum Toxin- A, ITB- Intrathecal Baclofen, SDR- Selective Dorsal Rhizotomy.

† For intrathecal baclofen, only GMFCS IV and V were analyzed with IV as the reference.

.^*^ *p* < 0.05, ^**^ *p* < 0.01, ^***^ *p* < 0.001

**Table 4:** Exponentiated coefficients; 95% confidence intervals in brackets of the occupational therapy related treatment modalities

Outcome variables (yes/no)			
Independent variables (reference group)	Orthoses in the upper extremities	BTX- A in upper extremities	Occupational therapy interventions
Place of birth (outside of the Nordic countries)	0.86	0.74	0.79
	[0.62,1.20]	[0.46,1.17]	[0.59,1.04]
Gender (Female)	1.11	0.90	0.85
	[0.90,1.37]	[0.68,1.20]	[0.71,1.01]
place of birth X gender	0.97	1.18	1.36
	[0.59,1.61]	[0.59,2.39]	[0.89,2.08]
Age	1.04^***^	1.02	0.94^***^
	[1.02,1.06]	[0.99,1.05]	[0.92,0.96]
Manual Ability Classification System (MACS I)			
II	9.36^***^	11.92^***^	2.92^***^
	[6.10,14.38]	[6.09,23.34]	[2.32,3.68]
III	18.41^***^	23.86^***^	4.29^***^
	[11.44,29.65]	[11.70,48.63]	[3.23,5.70]
IV	31.10^***^	25.52^***^	4.073^***^
	[18.10,53.42]	[11.57,56.31]	[2.86,5.80]
V	38.95^***^	25.00^***^	5.591^***^
	[21.44,70.78]	[10.53,59.36]	[3.63,8.62]
Gross Motor Function Classification System (GMFCS I)			
II	0.50^***^	0.56^**^	0.90
	[0.36,0.69]	[0.37,0.86]	[0.70,1.15]
III	0.19^***^	0.16^***^	1.10
	[0.12,0.31]	[0.079,0.34]	[0.82,1.48]
IV	0.35^***^	0.54^*^	1.41^*^
	[0.24,0.52]	[0.33,0.87]	[1.03,1.93]
V	0.53^**^	0.60	1.35
	[0.33,0.86]	[0.32,1.11]	[0.89,2.05]
Observations	3397	3397	3095

Relationship, presented as odds ratios with 95% confidence intervals in brackets, of the occupational therapist reported outcome variables and gender, adjusted for place of birth, age, Manual Ability Classification System (MACS) and Gross Motor Function Classification System (GMFCS). BTX-A- Botulinum toxin- A. ^*^ *p* < 0.05, ^**^ *p* < 0.01, ^***^ *p* < 0.001

Girls were significantly more likely than boys to have spinal braces. The interaction between place of birth and gender was significant, meaning that children born outside of the Nordic countries were significantly less likely to have spinal braces. The odds ratios for spinal braces increased significantly with age, as did the use of spinal brace by GMFCS level; those at lower GMFCS level (i.e., better gross motor function) had lower odds of having spinal braces. No significant differences were found between genders on BTX-A in the lower extremities. Higher spasticity scores were statistically significantly associated with higher odds of having received BTX-A.

Although girls were less likely to have ITB, it did not reach statistical significance. Being born outside of the Nordic countries was significantly associated with lower odds of having ITB. The odds of having ITB increased with age and those at GMFCS V were more likely than those at GMFCS IV to have ITB. There was no statistically significant difference in orally distributed spasticity reducing medications by gender. The OR of receiving spasticity reducing medication (orally) increased with age, those at higher GMFCS were significantly more likely to receive spasticity reducing medication, and higher spasticity scores were associated with higher odds of receiving spasticity reducing medication.

Girls were significantly less likely to have undergone SDR than boys. Participants at GMFCS II, III and IV had significantly higher odds of having undergone SDRs compared to those at GMFCS I. Furthermore, lower spasticity scores were significantly associated with having undergone SDR. The OR of having an assistive device that facilitated standing increased with GMFCS level (i.e., lower gross motor function). Also, higher spasticity scores were associated with higher odds of having an assistive device that facilitated standing.

Orthoses in the upper extremities were not significantly associated with gender. It was, however, positively associated with age in that the odds of having orthoses for the upper extremities increased with age. Furthermore, the odds of having orthoses for the upper extremities increased with MACS level, and seemed to decrease with GMFCS level. Receiving BTX-A in the upper extremities was positively associated with MACS and younger children were significantly more likely to have received OT treatment outside of CPUP or in the last year. The odds also increased with MACS level, meaning that those with more affected manual abilities were more likely to receive extra OT treatment. Those at GMFCS IV were more likely to receive additional OT treatments than those at GMFCS I.

## Discussion

In this study, the aim was to assess if the likelihood of receiving certain treatments and interventions were associated with the gender of the child. We also assessed if the gender of the child interacted with whether a person was born within or outside of the Nordic countries in terms of treatment and interventions received. The study population was found representative and comparable to other total population estimates of CP. Distributions of GMFCS-levels reported in Norway [19], Scotland [33], Australia [21] and the United States [20] are similar. However, the use of different treatment options are depending on treatment traditions and availability. There is a lack of other population-based studies for comparison of rates of the different treatments, which makes it difficult to make comparison across nations.

Girls were significantly more likely to have spinal braces, if they were not born outside of the Nordic countries. This finding is in line with recent research where girls with CP were slightly more likely to have scoliosis than boys with CP [34], and as such this appear to be a justified difference in treatment between the genders. However, why children born outside of the Nordic countries would be significantly less likely to have spinal braces is more difficult to explain. It is not known whether those born outside of the Nordic countries had recently immigrated to Sweden or moved to Sweden at a young age. It is possible that this finding might be explained by children born outside of the Nordic countries having participated in CPUP for a shorter period of time, and in time they might be offered spinal braces, if indicated. However, participants born outside of the Nordic countries were also less likely to have ITB. The same hypothesized explanation may help explain this finding. Importantly, ITB treatment requires that the individual stay in a country where tertiary care is available in order to fill the pump with Baclofen, approximately every third month. If the medication was to run out, the individual would suffer from severe and life-threatening withdrawal symptoms. Close to all children residing in Sweden with CP are included in CPUP. However, some children may not be permanent residents. Consequently, providers might be hesitant to recommend ITB for children if there is no guarantee that they will be able to stay in the country or be able to refill the medication. It was not analyzed how many of the children born outside of the Nordic countries were permanent Swedish residents. It is also possible that, for some reason, families from cultures other than the mainstream culture in the Nordic countries might prefer to not receive ITB as treatment for spasticity, or possibly that they have not understood what the treatment was for, and thus been hesitant to agree to the tretament. It needs to be investigated further if that is indeed the case or if there are other underlying reasons for this finding.

Girls were less likely to have undergone SDR, which, to the best of our knowledge, has not previously been reported in population-based studies. While it would be useful to compare these results to international studies, it is difficult to do. Population-based rates of SDR in different populations are very limited, many centers or clinics report outcome data from their units. A recent North American registry study reported rates of SDR in 0.1% in their sample of individuals receiving Medicaid, which is lower than 3% in the Swedish population in the present paper. It is possible that the rates of SDR would have been substantially higher if children on private insurances had been included. SDR is, to some extent, a controversial surgery and healthcare providers have varying opinions in terms of its usefulness [35]. That does not explain why more boys than girls have undergone the procedure. In the current study, a number of variables, such as GMFCS and MACS levels were adjusted for. However, according to a large population-based study as well as a recent Cochrane critical review, no statistically significant differences have been found on GMFCS and MACS-levels by gender [12, 36]. It is possible that there were other variables (e.g., subtypes of CP) not included in the current study that differed between genders and that these would have helped explain the results observed. Gender differences have been noted according to subtypes with boys presenting more often with spastic bilateral [12, 36] and dyskinetic types of CP, and girls more often with ataxic diplegia [36]. It is possible that the differences in subtypes help explain why boys are more likely to undergo SDR.

In a previous population based study on the use of BTX-A, significantly more boys (28%) than girls (23%) received BTX-A (OR = 1.25 [95% CI 1.05–1.48]) [22]. Our current research did not find support for this finding after controlling for GMFCS level, age and spasticity. Moreover, contrary to previous research [23] we did not find a gender difference in terms of PT and OT interventions received. Degerstedt et al. included data from a specific region of Sweden whereas the current study included data from all of Sweden. Furthermore, we did not assess whether the length and duration of interventions differed by gender, and it is possible that there would have been differences by gender had we done that.

There were several limitations to this study. It was cross-sectional in nature and cause and effect cannot be determined. Furthermore, we primarily used yes and no screening items. While dichotomous variables have their advantages, it is also possible to miss important nuances, which might be difficult to distinguish if trying to assess unconscious or conscious bias. Strengths of this research include that it was based on a total population, thus reducing the risk of selection bias and increasing generalizability of findings.

## Conclusion

The study showed more spinal brace use in girls, and girls were less likely to receive SDR than boys, also children born outside of the Nordic countries received less ITB. Other treatments and interventions registered in the CPUP PT and OT assessment forms were associated with age, levels of spasticity, GMFCS and MACS but were not associated with gender or place of birth.

## Data Availability

Data used in this study are stored at the National Quality Registry CPUP (http://rcsyd.se/anslutna-register/cpup). Data are not publicly available and permission to extract data can be obtained from the registry holder.

## Abbreviations

BTX-A: Botulinum toxin- A
CI: Confidence Interval
CP: Cerebral Palsy
CPUP: Swedish national cerebral palsy registry and follow-up program
GMFCS: Gross Motor Function Classification System
ITB: Intrathecal Baclofen
MACS: Manual Ability Classification System
OR: Odds Ratio
OT: Occupational Therapy
PT: Physiotherapy
SD: Standard Deviation
SDR: Selective Dorsal Rhizotomy
